# Plasma Expression and *In Silico* Functional Analysis of miR-106b-5p and miR-185-5p in Chronic Heart Failure

**DOI:** 10.3390/biom16050702

**Published:** 2026-05-11

**Authors:** Phuong Anh Huynh, Bao-Quoc Vu, Vu Hoang Vu, Minh Hoang, Diem My Vu

**Affiliations:** 1 Center for Molecular Biomedicine, University of Medicine and Pharmacy at Ho Chi Minh City, Ho Chi Minh City 700000, Vietnam; 2 Department of Internal Medicine, University of Medicine and Pharmacy at Ho Chi Minh City, Ho Chi Minh City 700000, Vietnam; 3 Cardiovascular Center, University Medical Center Ho Chi Minh City, Ho Chi Minh City 700000, Vietnam; 4 Pasteur Institute of Ho Chi Minh City, Ho Chi Minh City 700000, Vietnam

**Keywords:** plasma miR-106b-5p, plasma miR-185-5p, heart failure

## Abstract

Heart failure (HF) is one of the largest contributors to disease burden and healthcare expenditure worldwide. Countless studies have shown that microRNAs (miRNAs) are pivotal regulators of heart homeostasis and promising biomarkers for the diagnosis and management of HF. Among the reported miRNAs, miR-106b-5p and miR-185-5p have been implicated in various cardiovascular diseases through involvement in cardiac injury, fibrosis, and cell survival pathways. Although cellular functions of miR-106b-5p and miR-185-5p have been investigated intensively, their circulating levels remain largely elusive in patients with HF. This study examined expression levels of plasma miR-106b-5p and miR-185-5p by quantitative reverse transcription PCR (RT-qPCR) in a study cohort comprising 41 chronic HF patients and 41 matched, non-HF subjects. Bioinformatic analysis was conducted for miR-106b-5p and miR-185-5p to discover their potential target genes, biological functions, and association with cardiovascular-related clinical phenotypes. Chronic HF patients presented a significant increase in plasma miR-106b-5p and miR-185-5p levels. Diverse expressive patterns of miR-106b-5p and miR-185-5p were observed in different types and functional classes of HF. A positive correlation between plasma miR-106b-5p and miR-185-5p was also identified. *In silico* analysis suggested that many genes related to cell proliferation and metabolic pathways were shared targets of miR-106b-5p and miR-185-5p. Our study reveals dysregulation of plasma miR-106b-5p and miR-185-5p in patients with chronic HF that may contribute to the pathological course of this disease.

## 1. Introduction

Heart failure (HF) is a complex clinical syndrome which imposes a high burden of morbidity, hospitalization, and mortality worldwide [1,2]. It can arise as an end stage of most cardiovascular disorders such as myocardial infarction (MI), coronary artery disease, hypertension, and cardiomyopathy [3,4]. An increase in HF prevalence has been predicted due to population ageing and the increased prevalence of comorbid diseases such as obesity, diabetes, and hypertension [5,6]. Current practice in HF diagnosis mainly relies on clinical manifestations, cardiac imaging, and blood natriuretic peptide levels, and faces significant challenges due to overlapping symptoms of different HF types [7,8]. Therefore, enhanced understanding of HF’s underlying mechanisms will provide advanced strategies to improve the current approaches in disease diagnosis and patient classification.

In recent years, countless studies have demonstrated crucial roles of microRNAs (miRNAs) in the formation and normal development of the heart [9,10]. miRNAs are a class of short, single-stranded, non-coding RNAs involved in post-transcriptional regulation of gene expression [11,12]. Notably, a single miRNA molecule can influence the expression of multiple target genes, thereby simultaneously modulating various cellular and molecular processes [13]. miRNA dysregulation has been observed in numerous pathological processes of the heart and vascular system, such as cardiac hypertrophy, apoptosis, and fibrosis [14]. Additionally, different studies have reported the stable presence of certain miRNAs in body fluids and their altered expression in response to acute and chronic states of specific disease contexts, including HF, making them ideal candidate biomarkers [15].

In recent research, we performed comprehensive profiling of circulating miRNAs using next-generation sequencing in small cohorts of HF patients and non-HF participants that resulted in the identification of distinct miRNAs associated with HF, including miR-106b-5p and miR-185-5p [16]. In the cardiovascular context, miR-106b-5p plays a multifaceted role in modulating cardiac injury, inflammation, and remodelling [17]. miR-185-5p has been associated with the development of cardiac hypertrophy, apoptosis, and myocardial fibrosis [18]. Although the dysregulation of miR-185-5p was observed in right ventricular or dilated cardiomyopathy [19,20], circulating levels of miR-106b-5p and miR-185-5p in HF patients remain largely elusive. Considering their roles in pathological processes of the heart and our previous findings, this study examined expression patterns of plasma miR-106b-5p and miR-185-5p in larger cohorts of HF and non-HF patients. We also assessed the relationship between miRNA levels and cardiac function parameters. In addition, bioinformatic analysis was performed to elucidate potential functions and clinical phenotypes of miR-106b-5p and miR-185-5p.

## 2. Materials and Methods

### 2.1. Study Design and Participant Selection

Our study included 41 HF patients and 41 non-HF cases who were consecutively recruited from the University Medical Center Ho Chi Minh City, Vietnam, from June 2024 to December 2024. The inclusion criteria were: age > 18 years; stable HF for at least three months regardless of the cause and left ventricular ejection fraction (LVEF); and New York Heart Association (NYHA) classification of HF, class ≥ II. The exclusion criteria were as follows: acute myocardial infarction, chronic renal failure, known malignancy, surgery or transplantation within three months before enrollment, autoimmune disease, pregnancy and breastfeeding. Non-HF subjects were matched to HF cases by age (±5 years old) and gender.

### 2.2. Plasma Collection and miRNA Isolation

A total of 4 mL of venous blood samples was withdrawn from each study participant in an EDTA-anticoagulant tube (BD, San Jose, CA, USA). Plasma was collected from whole blood samples by centrifuging at 4400 rpm for 15 min. Following the centrifugation, plasma was then transferred into a new RNase/DNase-free tube and kept at −70 °C until subsequent analysis.

Total miRNAs were extracted from plasma by using a Hybrid-R^TM^ miRNA extraction kit (#325-150, GeneAll Biotechnology, Seoul, Republic of Korea) following the manufacturer’s instructions. Isolated miRNAs were then stored at −70 °C for further experiments.

### 2.3. Measurement of Plasma miR-106b-5p and miR-185-5p

Reverse transcription was performed using a miRCURY LNA^TM^ RT Kit (#339340, Qiagen, Hilden, Germany) following the manufacturer’s guidelines. miRNA expression was measured using a miRCURY LNA SYBR Green PCR Kit (#339345, Qiagen, Hilden, Germany) with a pre-designed miRCURY LNA miRNA PCR assay for miR-106b-5p and miR-185-5p (#339306, Qiagen, Hilden, Germany). miR-103a was used as the reference as previously described [21]. The reaction was performed with the following programme: 95 °C for 2 min, 45 cycles of 95 °C for 10 s and 60 °C for 30 s, then followed by melting curve analysis from 60 °C to 95 °C. A cycle threshold (Ct) value ≥ 40 was considered undetermined. The relative miRNA expression levels were calculated using the formula 2^−∆Ct^. All experiments were performed in duplicate.

### 2.4. Target Gene Prediction and Pathway Enrichment Analysis

The target genes of miR-106b-5p and miR-185-5p were predicted using the miRTargetLink 2.0 database [22]. FunRich version 3.1.4, a publicly accessible software for functional enrichment and interaction network analysis, was used to determine clinical phenotypes associated with cardiovascular diseases of identified target genes [23]. Strongly predicted target genes of miR-106b-5p and miR-185-5p were subsequently selected for pathway and Gene Ontology enrichment analysis using g: Profiler version e113_eg59_p19 [24].

### 2.5. Statistical Analysis

Continuous variables were expressed as mean ± standard deviation (SD) when normally distributed or median with interquartile range (IQR) when non-normally distributed, and categorical variables were expressed as percentages unless otherwise stated. The Shapiro–Wilk test was used to assess the distribution for each parameter. Continuous variables were compared using Student’s *t*-test or the Mann–Whitney test, and Fisher’s exact test was used for the comparison of categorical variables. For the miRNA expression, the Mann–Whitney U and Kruskal–Wallis tests, followed by Dunn’s correction for multiple comparisons, evaluated the significance of differences between study groups. The associations of miRNA expression levels were analyzed by Spearman’s rank correlation. A *p*-value of <0.05 was considered statistically significant. All analyses were performed with GraphPad Prism 8 (GraphPad Software, Boston, MA, USA).

## 3. Results

### 3.1. Baseline Characteristics and Drugs Used by the Study Population

The baseline characteristics of all study participants are summarized in Table 1. Patients’ age ranged from 26 to 87 years in the HF group, and from 30 to 84 years in the non-HF group. Male patients were predominant at 60.9% of participants. There were no statistical differences between HF patients and non-HF subjects in terms of age, gender, BMI, and heart rate. Other variables, such as systolic/diastolic blood pressure and % LVEF, were significantly lower in the HF group (Table 1). As expected, HF patients were more frequently found with cardiovascular risk factors and drugs used than their non-HF counterparts (Table 1).

### 3.2. Plasma miR-106b-5p and miR-185-5p Expressions and Cardiac Functional Indexes

**Figure 1:**
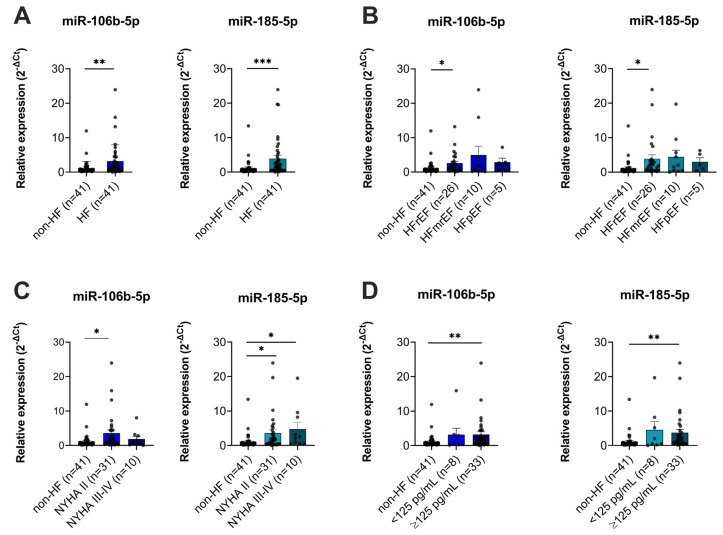
Plasma miR-106b-5p and miR-185-5p expressions in chronic HF patients and non-HF subjects. (**A**) Relative expression of plasma miR-106b-5p and miR-185-5p in HF and non-HF groups. Expression levels related to: (**B**) left ventricular ejection fraction classification, (**C**) NYHA functional class, and (**D**) NT-proBNP level. The relative expression was calculated as 2^−∆Ct^. Bar graphs indicate mean ± SEM. The differences were analyzed by Student’s *t*-test or the Kruskal–Wallis ANOVA test; (* *p* < 0.05, ** *p* < 0.01, and *** *p* < 0.001). HF: heart failure; non-HF: non-heart failure; HFrEF: heart failure with reduced ejection fraction; HFmrEF: heart failure with mildly reduced ejection fraction; HFpEF: heart failure with preserved ejection fraction; NYHA: New York Heart Association classification of heart failure.

The expression of plasma miR-106b-5p and miR-185-5p was then measured by RT-qPCR for the whole study population. Generally, compared to non-HF subjects, a significant increase in miR-106b-5p and miR-185-5p levels was observed in HF patients (Figure 1A).

In more detail, according to the LVEF value, HF can be divided into different types, such as HF with reduced ejection fraction (HFrEF), HF with mildly reduced ejection fraction (HFmrEF), or HF with preserved ejection fraction (HFpEF) [3]. Although the patients shared similar morbidity and mortality risks, a distinct pathophysiology was reported [25]. We obtained a significant increase in plasma levels of miR-106b-5p and miR-185-5p in the HFrEF subgroup. Indeed, insignificantly elevated expressions of these miRNAs were also detected in the HFpEF and HFmrEF groups (Figure 1B).

Since there was only one patient with NYHA class IV, we then classified HF cases into two functional groups, with 31 cases belonging to the NYHA II group, and the other 8 patients grouped into the NYHA III-IV group. Interestingly, as shown in Figure 1C, different patterns of plasma miR-106b-5p and miR-185-5p expressions were obtained. Whereas a significant upregulation of miR-106b-5p was detected in the NYHA II group, more severe HF cases presented a higher level of plasma miR-185-5p.

When stratifying HF patients based on their N-terminal prohormone of brain natriuretic peptide (NT-proBNP) level, our study cohort comprised 8 HF patients with NT-proBNP levels < 125 pg/mL and 33 cases with NT-proBNP values ≥ 125 pg/mL. Both of these groups showed an upregulation of miR-106b-5p and miR-185-5p (Figure 1D). Though HF patients with an NT-proBNP level < 125 pg/mL had a greater increase in these miRNA levels, a significant difference was solely confirmed in cases that had an NT-proBNP value ≥ 125 pg/mL.

### 3.3. Relationship Between Plasma miR-106b-5p and miR-185-5p and Cardiac Clinical Parameters

**Figure 2:**
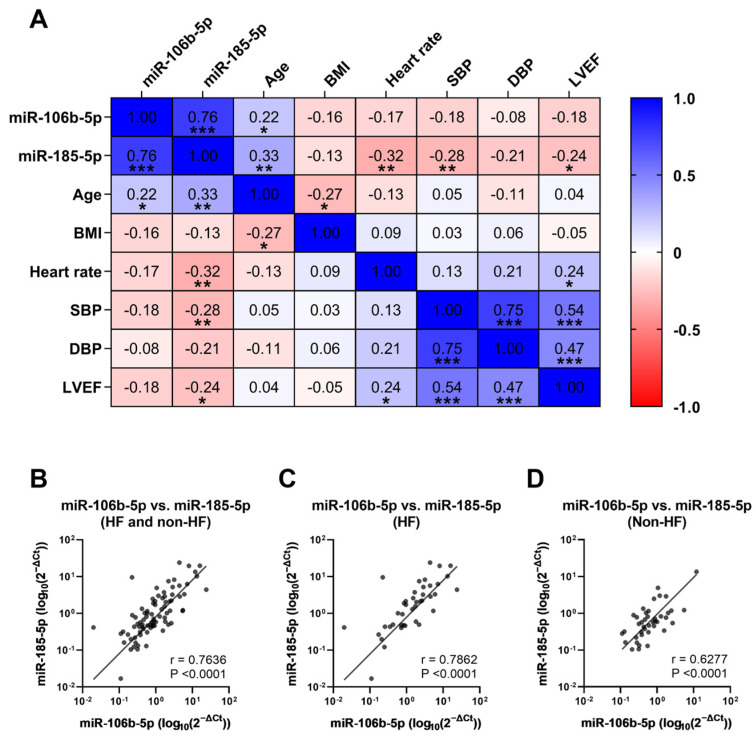
Relationship between plasma levels of miR-106b-5p and miR-185-5p and other clinical parameters. (**A**) Spearman correlation analyses were performed between miRNA expression and various clinical parameters. Positive correlation between plasma miR-106b-5p and miR-185-5p in (**B**) all study participants, (**C**) HF group, and (**D**) non-HF group. BMI, body mass index; SBP, systolic blood pressure; DBP, diastolic blood pressure; LVEF, left ventricular ejection fraction. (* *p* < 0.05, ** *p* < 0.01, and *** *p* < 0.001).

We next evaluated the possible correlation between miRNA levels and other parameters clinically relevant to cardiac function. When all study participants were taken into account, we found a positive correlation between the expressions of plasma miR-106b-5p and miR-185-5p (Figure 2A,B). A negligible relationship was detected for circulating amounts of miR-106b-5p and miR-185-5p and other factors (Figure 2A and Figure S1). Intriguingly, when analyzing samples within each group, a stronger relationship between plasma miR-106b-5p and miR-185-5p was observed in HF patients compared to non-HF subjects (Figure 2C,D).

### 3.4. Target Gene Prediction and Functional Analysis of miR-106b-5p and miR-185-5p

**Figure 3:**
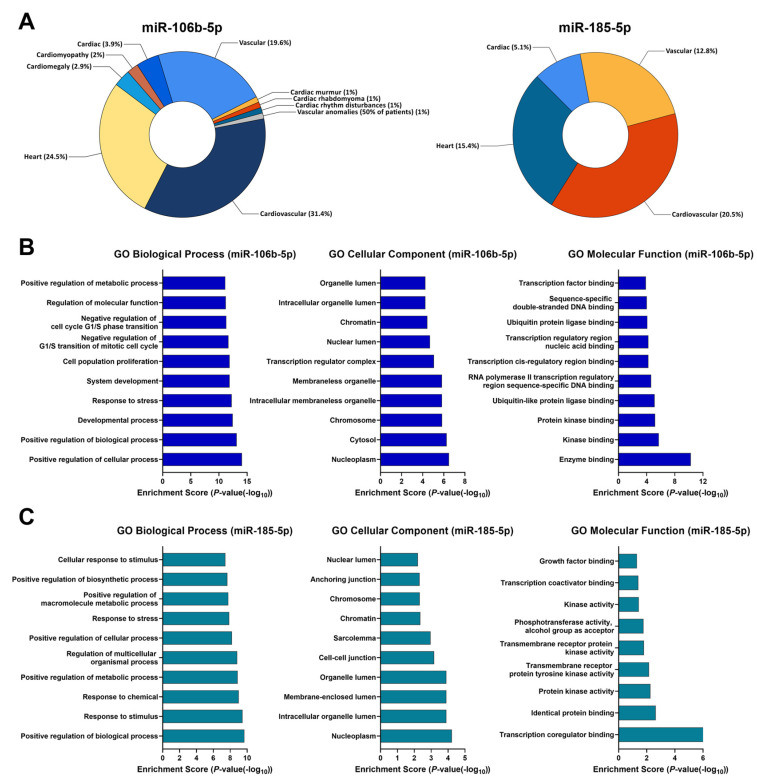
Target gene prediction and functional analyses of miR-106b-5p and miR-185-5p. (**A**) Pie plots represent cardiovascular-related clinical phenotypes that involve predicted target genes of miR-106b-5p and miR-185-5p. Percentages do not sum to 100% because only cardiovascular-related categories are included. (**B**,**C**) Gene Ontology analysis of strongly predicted genes of miR-106b-5p and miR-185-5p covering three domains: biological process, molecular function, and cellular component. The top 10 enrichment terms are reported for each bar graph.

We further examined the hypothetical functions of miR-106b-5p and miR-185-5p. Using miRTargetLink 2.0, we identified 1090 and 359 predicted target genes of miR-106b-5p and miR-185-5p, respectively (Table S1). Among those, 41 genes were confirmed as shared targets of these two miRNAs (Table S1). By querying FunRich, we explored whether predicted target genes could be linked to cardiovascular-related clinical phenotypes. Our results indicated that many of them were linked to heart and vascular diseases (Figure 3A).

In addition, we analyzed signalling pathways associated with 44 and 39 strongly predicted target genes of miR-106b-5p and miR-185-5p from the Gene Ontology for determining their functional outputs (Table S2). Our analysis showed the enrichment of different pathways associated with cell cycle, cell proliferation, and metabolic and stress responses (Figure 3B,C).

## 4. Discussion

The role of miRNAs in pathophysiology and their potential as a new class of biomarkers have received increasing attention in HF studies. Even though their cellular functions have been intensively characterized, the expression pattern and circulating level of many miRNAs remain to be determined. In this study, we delineated plasma expression of miR-106b-5p and miR-185-5p in patients with chronic HF. Our results showed that circulating miR-106b-5p and miR-185-5p were significantly upregulated in HF subjects. Moreover, we observed diverse expression patterns of each miRNA in different types and classes of HF. Further analysis revealed a close relationship between plasma levels of miR-106b-5p and miR-185-5p. Through target gene prediction and functional assessment, we demonstrated an involvement of miR-106b-5p and miR-185-5p in important pathways related to cell proliferation and responses to metabolic and stress stimuli, together with their association with cardiac-related clinical phenotypes.

miR-106b-5p is a member of the miR-106b family, which plays a major role in functional regulation of cell cycle progression, apoptosis, and proliferation [26,27]. It is involved in cardiovascular diseases by modulating the transforming growth factor-β (TGF-β) [28], mitogen-activated protein kinase (MAPK), and apoptotic pathways [29]. Several cell cycle regulators, such as E2F transcription factor 5 (*E2f5*), cyclin-dependent kinase inhibitor 1c (*Cdkn1c*), cyclin E1 (*Ccne1*), and *Wee1*, have been identified as direct targets of miR-106b-5p. miR-185-5p has been described as an important regulator of cardiac hypertrophy by directly targeting multiple genes within calcium-signalling pathways, including calcium/calmodulin-dependent protein kinase II delta (*Camk2d*), sodium calcium exchanger 1 (*Ncx1*), and nuclear factor of activated T cells 3 (*Nfatc3*) [30].

An increase in serum miR-106b-5p was reported in patients with coronary artery disease and myocardial ischemia, while a higher miR-185-5p level was documented in cardiac tissue of cardiomyopathy patients, correlating with increased profibrotic markers such as TGF-β1 and collagen I [18,31,32,33]. In this work, we observed significant overexpression of miR-106b-5p and miR-185-5p in the plasma of HF patients, similarly to previous observations in other cardiovascular disorders.

To assess whether the dysregulation of these miRNAs could relate to clinical features that characterize HF patients, we examined a possible association between plasma levels of miR-106b-5p and miR-185-5p and heart functional indices (LVEF types, NYHA class, and NT-proBNP). Based on LVEF classification, our data suggested that the overexpression of plasma miR-106b-5p and miR-185-5p was unrelated to a specific subtype of HF; though HFmrEF patients presented the greatest miRNA upregulation. Despite various degrees of overlap, much research has suggested a pathophysiological differences across HF subtypes [34]. For instance, a study by Tromp’s group showed that biological pathway changes were mainly related to cellular proliferation and metabolism in HFrEF, inflammation and extracellular matrix reorganization in HFpEF, and a mixture of both in HFmrEF [35]. Thus, these factors may contribute to the fluctuation of miRNA expression. NT-proBNP is a well-established biomarker used for risk stratification in HF, with its higher level normally indicating a more severe condition. When stratifying our patients by NT-proBNP, we found that plasma miR-106b-5p and miR-185-5p levels were either inversely or independently associated with an increase in NT-proBNP, respectively. Intriguingly, we obtained an opposite pattern of circulating miR-106b-5p and miR-185-5p expressions in different NYHA classes. Thus, these miRNA levels may be combined differently to discriminate certain types and classes of HF.

Numerous studies have independently reported the involvement of miR-106b-5p and miR-185-5p in cardiac fibrosis, inflammation, and apoptosis [36,37,38]. Examining miRNA’s relationship with clinical parameters, we obtained a positive correlation between plasma levels of miR-106b-5p and miR-185-5p, suggesting a potential interplay of these in the cardiovascular context. *In silico* prediction showed that various genes were co-targets of miR-106b-5p and miR-185-5p. Our observation was further supported by a high degree of biological functions shared by miR-106b-5p and miR-185-5p through GO analysis.

A major limitation of our study was a small sample size, with an unequal number of participants in divided subgroups, which may affect its generalizability. Larger, multicentre studies are required to consolidate and extend our findings. In addition, we did not assess the possible impacts of comorbidities and medications on miRNA expression. It is also important to perform time course measurements of miRNA levels, thereby clarifying their fluctuation over disease development and progression. Further in vitro and in vivo studies are required to support the hypothetical functions proposed by our analysis and to elucidate miRNA roles in the pathological course of HF.

## 5. Conclusions

Our study reveals the upregulation of plasma miR-106b-5p and miR-185-5p in chronic HF patients, with diverse expression patterns in types and functional classes of HF. We also identify shared target genes and biological functions of miR-106b-5p and miR-185-5p, suggesting an interplay between these miRNAs in the mechanism underlying the pathogenesis of HF.

## Figures and Tables

**Table 1:** Baseline characteristics of study participants.

Characteristics	Heart Failure Group (*n* = 41)	Non-Heart Failure Group (*n* = 41)	*p*-Value
Age (years)	57.9 ± 14.2	57.5 ± 13.3	0.879
Gender, *n* (% male)	25 (60.9%)	25 (60.9%)	1.000
Body mass index (Kg/m^2^)	24.1 ± 4.2	23.8 ± 3.1	0.657
Heart rate (beats/minute)	76.2 ± 13.8	81.7 ± 13.1	0.066
Systolic blood pressure (mm/Hg)	116.5 ± 19.3	136.8 ± 15.9	<0.0001 *
Diastolic blood pressure ^a^ (mm/Hg)	70 (60.0–75.5)	80 (70.0–84.5)	0.0004 *
NT-proBNP (pg/mL)	542 (157–1909)	n/a	-
% Left ventricular ejection fraction	37.1 ± 12.7	67.4 ± 6.3	<0.0001 *
NYHA class II, *n* (%)	31 (75.6)	0	-
NYHA class III, *n* (%)	9 (21.9)	0	-
NYHA class IV, *n* (%)	1 (2.4)	0	-
Clinical history and risk factors, *n* (%)			
Hypertension	37 (90.2)	32 (78.1)	0.226
Diabetes	13 (31.7)	7 (17.1)	0.198
Hyperlipidemia	32 (78.1)	18 (43.9)	0.003 *
History of myocardial infarction	17 (41.5)	0 (0.0)	<0.0001 *
Myocarditis	1 (2.4)	0 (0.0)	>0.999
Ischemic heart diseases	5 (12.2)	2 (4.9)	0.432
Coronary syndrome	23 (56.1)	1 (2.4)	<0.0001 *
Angina pectoris	2 (4.9)	0 (0.0)	0.494
Dilated cardiomyopathy	6 (14.6)	0 (0.0)	0.026 *
Medications, *n* (%)			
RAASi	37 (90.2)	22 (53.7)	0.0004 *
Betablockers	39 (95.1)	7 (17.1)	<0.0001 *
MRA	36 (87.8)	0	<0.0001 *
SGLT2i	39 (95.1)	0	<0.0001 *
Antiplatelets	28 (68.3)	5 (12.2)	<0.0001 *
Statins	34 (82.9)	19 (46.3)	0.00101 *
Calcium channel blockers	0	18 (43.9)	<0.0001 *
Furosemide	3 (7.3)	0	0.2407

* Statistical significance. ^a^ Mann–Whitney U test. NT-proBNP: N-terminal prohormone of brain natriuretic peptide; NYHA: New York Heart Association classification of heart failure; RAASi: renin–angiotensin–aldosterone system inhibitors; MRA: mineralocorticoid receptor antagonist; SGLT2i: sodium–glucose cotransporter-2 inhibitors.

## Data Availability

The data presented in this study are available in the results and Supplementary Material.

## Supplementary Materials

The following supporting information can be downloaded at: https://www.mdpi.com/article/10.3390/biom16050702/s1. Table S1. Predicted target genes of miR-106b-5p and miR-185-5p. Table S2. Strongly predicted target genes of miR-106b-5p and miR-185-5p. Figrue S1. Scatter plots of the relationship between plasma levels of miR-106b-5p and miR-185-5p and other clinical parameters.
