# Supplementary figures and images for: Plasma Expression and In Silico Functional Analysis of miR-106b-5p and miR-185-5p in Chronic Heart Failure
Source: Biomolecules. 2026 May 11;16(5):702. doi: 10.3390/biom16050702 (PMC13204167; doi:10.3390/biom16050702)

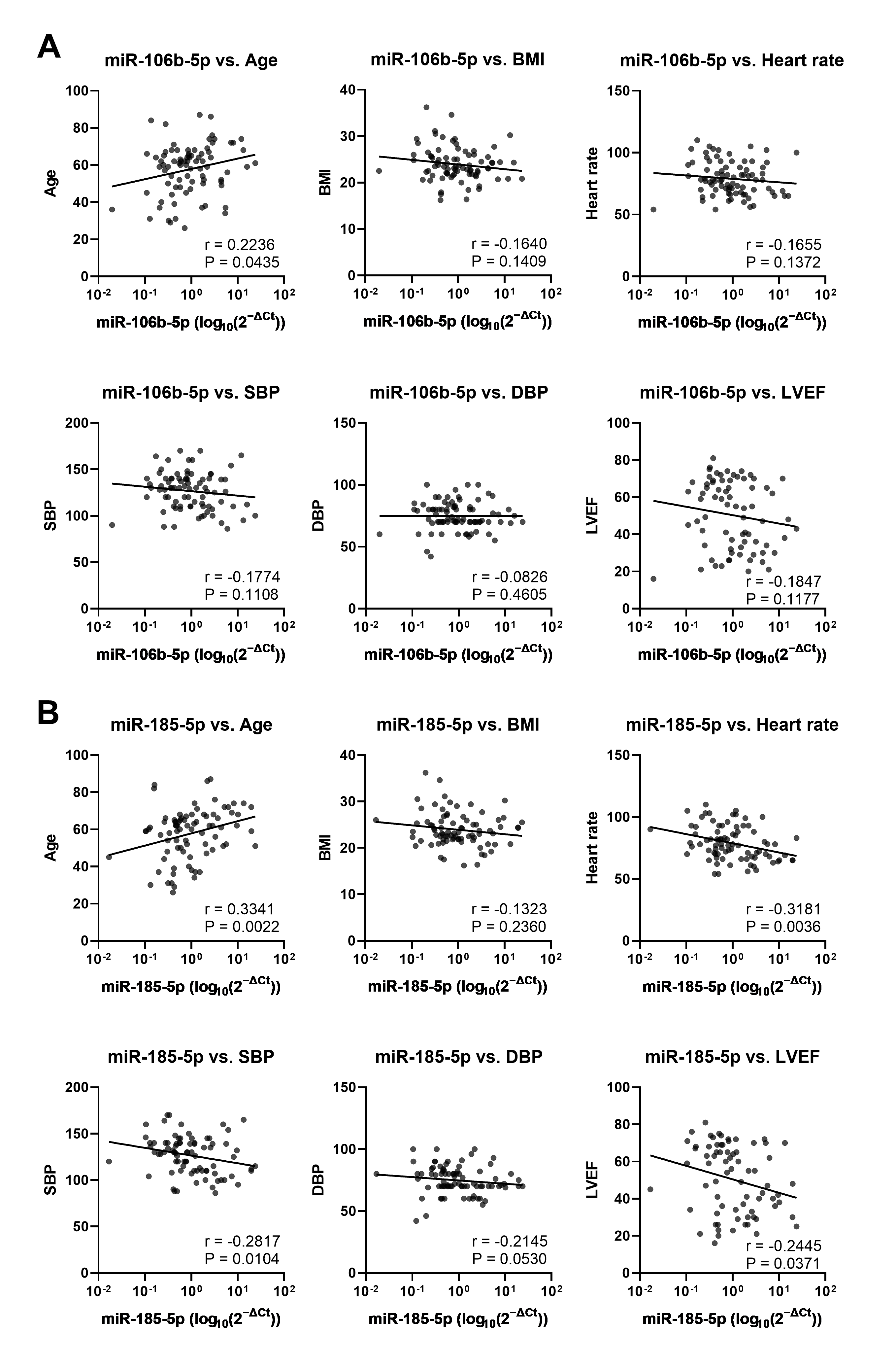

Supplement: Supplementary file 1 [file biomolecules-16-00702-s001.zip › FigS1_Done.tif]
